# A case report and literature review of primary distal renal tubular acidosis resulting from a mutation in *ATP6V0A4*

**DOI:** 10.3389/fped.2025.1685798

**Published:** 2025-11-27

**Authors:** Chenyang Chang, Hao Chen, Min Wang, Jingshan Chen, Yaomin Zou, Shaowen Hu, Kaiyuan Luo, Xingyu Rao, Huifang Zhu

**Affiliations:** 1 Pediatric Internal Medicine, Children’s Medical Center, First Affiliated Hospital of Gannan Medical University, Ganzhou, Jiangxi, China; 2 Pediatric Intensive Care Unit, Fuqing City Hospital of Fujian, Fuzhou, Fujian, China; 3 Neonatal/Pediatric Intensive Care Unit, Children’s Medical Center, First Affiliated Hospital of Gannan Medical University, Ganzhou, Jiangxi, China; 4 The First Clinical Medical College of Gannan Medical University, Ganzhou, Jiangxi, China; 5 Institute of Children’s Medical, First Affiliated Hospital of Gannan Medical University, Ganzhou, Jiangxi, China; 6 Ganzhou Key Laboratory of Immunotherapeutic Drugs Developing for Childhood Leukemia, Ganzhou, Jiangxi, China; 7 Basic Medical College of Gannan Medical University, Ganzhou, Jiangxi, China

**Keywords:** primary distal renal tubular acidosis, *ATP6V0A4*, missense mutation, case report, autosomal recessive

## Abstract

Primary renal tubular acidosis is an inherited disorder with heterogeneous clinical presentations, including chronic metabolic acidosis, electrolyte imbalances (e.g., hypokalemia), skeletal abnormalities, and urinary tract symptoms. We report a noteworthy case of a 7-month-old infant who presented with recurrent vomiting and hypokalemia. Laboratory investigations confirmed hypokalemia, metabolic acidosis with hyperchloremia, and inappropriately alkaline urine, leading to a strong suspicion of primary renal tubular acidosis. Genetic analysis by whole-exome sequencing identified a novel homozygous missense mutation (c.1418C > T) in the *ATP6V0A4* gene on chromosome 7 in the proband. This specific variant is exceptionally rare and is associated with a severe, atypical phenotype manifesting in early infancy. This case expands the known mutational and phenotypic spectrum of *ATP6V0A4*-related renal tubular acidosis. Our findings aim to enhance the understanding of this disease by correlating the clinical course with its genetic etiology, thereby establishing a molecular basis for precise etiological diagnosis, informed genetic counseling, and prenatal diagnosis.

## Introduction

Primary distal renal tubular acidosis (dRTA) is a rare genetic disorder characterized by impaired hydrogen ion secretion in the distal renal tubules and an inadequate capacity of renal tubules to generate sufficient titratable acids and ammonium (1, 2). The primary characteristics include metabolic acidosis with a normal anion gap, hypokalemia, hyperchloremia, renal manifestations such as hypercalciuria, abnormal alkaline urine, renal calcification, and proteinuria; as well as extrarenal manifestations including hearing loss, enamel hypoplasia, growth retardation, and anemia (3). Primary dRTA is associated with both autosomal dominant (AD) and/or autosomal recessive (AR) inheritance patterns (4). *SLC4A1*, *ATP6V1B1*, *ATP6V0A4*, *FOXI1*, *WDR72*, and *ATP6V1C2* are implicated in the pathogenesis of dRTA (5–9). Variations in the *ATP6V0A4* gene are associated with AR inheritance and are frequently linked to sensorineural hearing loss (9).

We present a rare case of primary dRTA resulting from a homozygous mutation in the *ATP6V0A4* gene. By analyzing the clinical and genetic characteristics of the patient, along with a comprehensive literature review, we aim to enhance clinicians' understanding of dRTA to minimize instances of missed or misdiagnosis.

## Case description

We report the case of a female child aged 7 months and 20 days who was hospitalized in our hospital. Vomiting began more than 20 days before admission, occurring without any identifiable trigger. 5 days before admission, following the onset of generalized weakness, paralysis, and an inability to lift the head—accompanied by abdominal distension and constipation—the child was admitted to an outside hospital. It is noteworthy that significant polyuria was absent. The initial therapeutic regimen consisted of cefotaxime for empirical antimicrobial coverage and potassium chloride to correct hypokalemia. Following a reduction in the dosage of potassium chloride, the child once again exhibited hypokalemia and diminished appetite, prompting her transfer to our hospital for further management. The outpatient department recommended hospitalization due to “hypokalemia”. The child was the second born of the family. The child was delivered at term via cesarean section with a birth weight of 3.2 kg. There was no parental consanguinity, and both parents as well as the patient's sister exhibited an unremarkable phenotype without significant abnormalities. Vital signs on admission were as follows: temperature 36.2 °C, heart rate 152 beats per minute, respiratory rate 38 breaths per minute, and blood pressure 98/52 mmHg. The patient was alert and conscious, in fair general condition, with a flat anterior fontanelle. Skin turgor was normal. A comprehensive assessment of the cardiopulmonary and abdominal systems indicated no notable abnormalities. The muscle strength and tension in the limbs were found to be within normal limits. The physiological reflex was evident, whereas the pathological reflex was not elicited.

Preliminary laboratory analyses revealed arterial blood gas results indicative of metabolic acidosis (PH 7.30, base excess −13.90), without significant electrolyte imbalances (oral potassium chloride administered prior to admission) (see Table 1). Urine pH was recorded at 7.0, specific gravity at 1.003 (see Table 1), along with elevated aldosterone levels (3,042.77 pg/mL) and increased renin activity (70.686 ng/mL/h). The complete blood count was largely within normal ranges, and no significant abnormalities were detected in thyroid function, cortisol levels, adrenocorticotropin, liver function tests, renal function parameters, or blood glucose concentrations. The electrocardiogram (ECG) revealed the presence of sinus tachycardia. No significant abnormalities were observed in the ultrasonography of both kidneys and renal arteries, cardiac ultrasound, or adrenal CT imaging. Following admission, potassium supplementation was withheld for a duration of 24 h. Subsequent re-evaluation of blood gas and electrolyte levels revealed a normal anion gap, hyperchloremic metabolic acidosis, and serum potassium measured at 3.79 mmol/L (reference range: 4.2–5.9 mmol/L) (see Table 2).

**Table 1:** Relevant important laboratory tests upon admission.

Parameter	Result	Unit
Serum electrolytes		
Potassium	4.51	mmol/L
Sodium	139.30	mmol/L
Chlorine	112.80	mmol/L
Calcium	2.56	mmol/L
Phosphorus	1.19	mmol/L
Arterial blood gas		
PH	7.30	
PO_2_	116.00	mmHg
PCO_2_	21.00	mmHg
HCO_3_^−^	10.30	mmol/L
Base excess	−13.90	mmol/L
O_2_ saturation	98.00	%
Urinalysis		
Color	Pale yellow	
PH	7.00	
Specific gravity	1.003	
White blood cells (manual microscopy)	4–8	/HP
Urine protein value	Negative	

**Table 2:** Subsequent investigation of laboratory findings.

Parameter	Result	Unit
Serum electrolytes		
Potassium	3.79	mmol/L
Sodium	141.90	mmol/L
Chlorine	110.90	mmol/L
Calcium	2.76	mmol/L
Phosphorus	1.36	mmol/L
Venous blood gas		
PH	7.31	
PO_2_	34.00	mmHg
PCO_2_	36.00	mmHg
HCO_3_^−^	18.10	mmol/L
Base excess	−7.60	mmol/L
O_2_ saturation	59.00	%

Based on the clinical presentation, laboratory findings, and imaging studies, dRTA was highly suspected; therefore, peripheral blood samples from the parents and the child were submitted for genetic testing.

Genetic testing results indicated the presence of a homozygous mutation, c.1418C > T, in the *ATP6V0A4* gene located on chromosome 7 of the child. The genetic characteristics aligned with the principles of AR inheritance. Based on the American College of Medical Genetics and Genomics (ACMG) variant classification, c.1418C > T may represent a potential pathogenic variant.

Upon discharge, the patient was administered spironolactone tablets at a dosage of 4 mg once daily and potassium citrate granules at a dosage of 0.24 g twice daily. In infants and young children, arterial blood gas, renal function, electrolyte levels, alkaline phosphatase, and urinalysis should be re-evaluated every 3–4 months (10). In stable older children, this assessment should occur every 6–12 months (10). Additionally, a follow-up ultrasound of the urinary system is recommended every 6–12 months. Each follow-up appointment should also include measurements of height and weight.

This study received approval from the Ethics Committee of the First Affiliated Hospital of Gannan Medical University (LLSC-2024 No. 264), and informed consent was obtained from the child's guardian.

## Discussion

**Figure 1:**
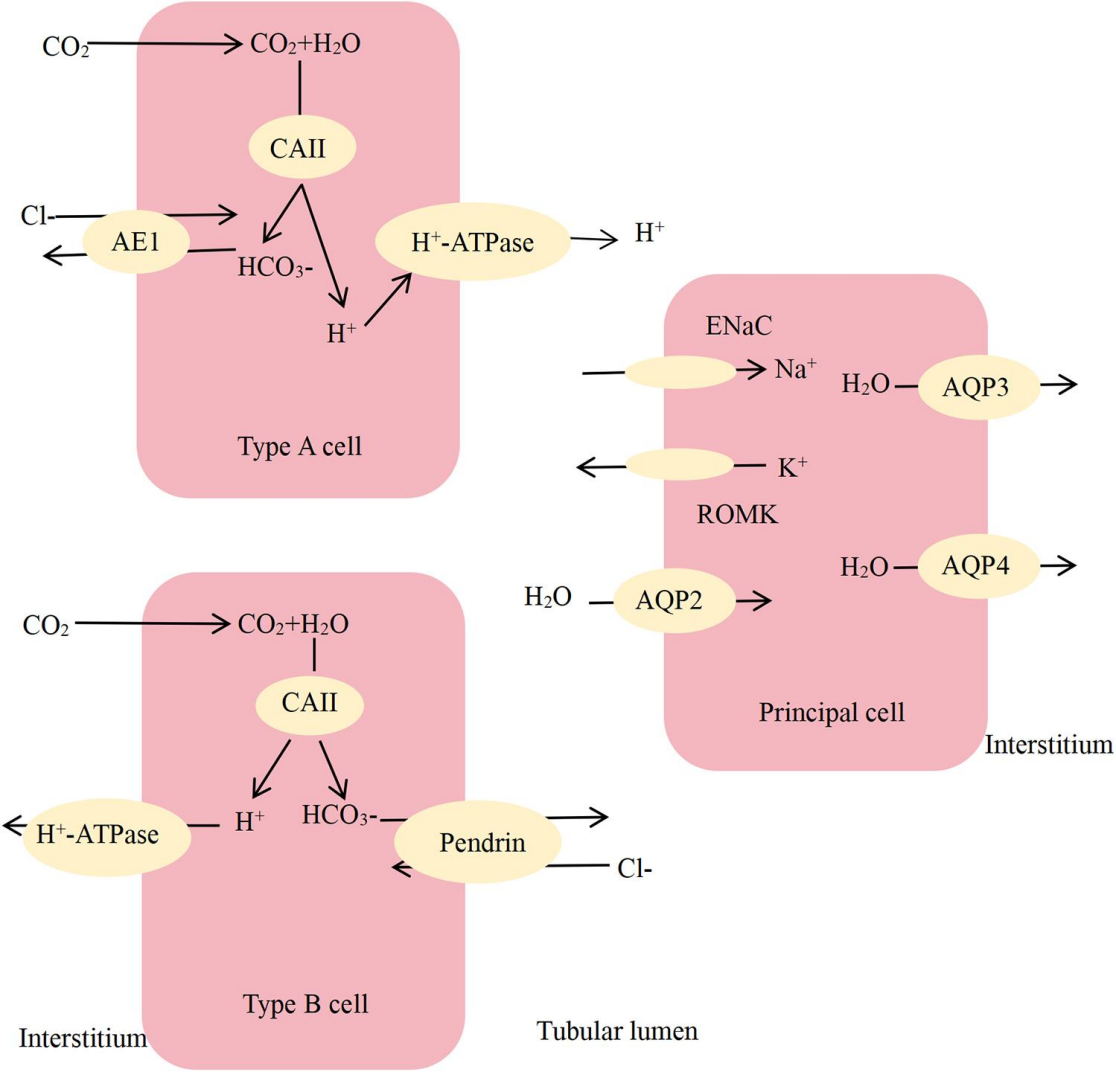
Schematic diagram of principal cells and various types of intercalated cells. CAII, carbonic anhydrase II; AE1, anion exchange protein 1; ENaC, epithelial sodium channel; AQP2, aquaporin 2; ROMK, renal outer medullary K + -channel; AQP3, aquaporin 3; AQP4, aquaporin 4.

In the distal renal tubules and collecting ducts, there exist two distinct types of cells: principal cells and intercalated cells (Figure 1). The principal cells facilitate the reabsorption of NaCl and H_2_O, concurrently secreting potassium ions. Conversely, intercalated cells predominantly secrete hydrogen ions while also contributing to potassium reabsorption. The epithelial sodium channel (ENaC), aquaporin 2 (AQP2), and ATP-sensitive inward rectifier potassium channel 1, commonly known as the renal outer medullary K + -channel (ROMK), are predominantly localized in the apical membrane of principal cells (11). Conversely, aquaporin 3 (AQP3) and aquaporin 4 (AQP4) are primarily located within the basement membrane (11). The intercalated cells located in the collecting duct are categorized into three distinct types: type A, type B, and non-type A/non-type B cells. Type A cells primarily participate in the secretion of hydrogen, whereas type B cells predominantly engage in the secretion of bicarbonate (11). Type A cells facilitate bicarbonate reabsorption through anion exchange protein 1 (AE1, also referred to as SLC4A1) located on the basement membrane, while hydrogen ions are secreted via H + -ATPases situated on the apical membrane (1, 12). Type B cells secrete bicarbonate via Pendrin located on the apical membrane, while reabsorbing hydrogen ions through H + -ATPases situated on the basolateral membrane (13). The initiation of primary dRTA is attributed to modifications in genes that encode or regulate the channels responsible for urine acidification within the distal renal tubules and collecting ducts. Mutations in the *SLC4A1*, *ATP6V1B1*, *ATP6V0A4*, and *ATP6V1C2* genes, which encode the AE1 protein, the B1 subunit of H + -ATPase, the A4 subunit of H + -ATPase, and the C2 subunit of V-type H + -ATPase, respectively, disrupt hydrogen ion secretion and are associated with the development of dRTA (Supplementary Table S1) (6, 14–16). Genetic variants in *SLC4A1*, *ATP6V1B1*, and *ATP6V0A4* are responsible for approximately 70% of cases of dRTA (17). The mutations observed in these genes encompass nonsense mutations, missense mutations, frameshift mutations, and splice site alterations. The *SLC4A1* gene is implicated in disease through both AD and AR inheritance (18, 19), while the *ATP6V1B1*, *ATP6V0A4*, and *ATP6V1C2* genes are associated with AR disorders (6, 15, 20). The onset of dRTA in children with AR inheritance generally manifests at an early age, frequently presenting during infancy. This condition tends to be relatively severe and can result in impaired growth and development, as well as renal insufficiency. This study reports that the affected children exhibit AR inheritance, with a pathogenic variant identified as c.1418C > T in the *ATP6V0A4* gene. Additionally, other mutations within the *ATP6V0A4* gene have also been observed, including p.W745X, c.2257 + 1G > A, c.2257 + 1G > A, c.722 + 5G > A, and c.1631C > T (21–23).

The diagnosis of primary dRTA should be established through a comprehensive synthesis of clinical manifestations and genetic testing results. In this study, the subject was a 7-month-old infant presenting with recurrent vomiting and hypokalemia. The clinical presentation was characterized by recurrent vomiting, progressive generalized weakness and paralysis, inability to lift the head, abdominal distension, and constipation. Laboratory tests indicated multiple episodes of hypokalemia, paradoxial alkaline urine, metabolic acidosis, hyperchloremia, and secondary aldosteronism. The clinical manifestations and auxiliary examinations were in accordance with the characteristics of primary dRTA. The results of the genetic testing indicated that *ATP6V0A4* harbored a potentially pathogenic missense mutation, thereby further substantiating the diagnosis of dRTA.

**Figure 2:**
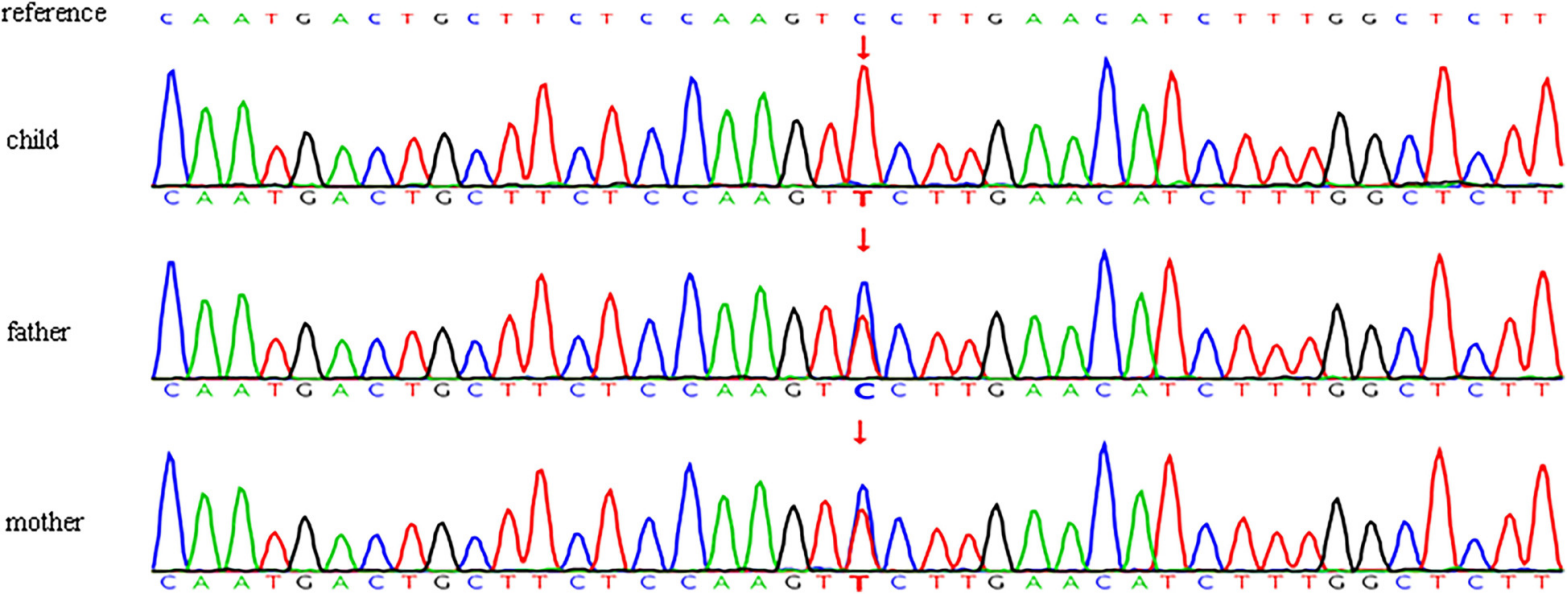
Schematic representation of the validation results obtained through sanger sequencing of *ATP6V0A4* variants in the child and her parents.

Peripheral blood samples from the child and their parents were dispatched to a laboratory in Beijing for whole-exon high-throughput sequencing. Genomic DNA sequencing identified putative pathogenic variants that may elucidate the patient's phenotype. A mutation in the coding region of *ATP6V0A4* at base 1,418, changing from C to T, resulted in an amino acid alteration at position 473, where serine was substituted by phenylalanine (NM_020632.3: C.1418C > T: p.S473F), as illustrated in Table 3. The c.1418C > T homozygous mutation in the ATP6V0A4 gene was accompanied by a paternal heterozygous mutation and a maternal heterozygous mutation, indicating mutations inherited from both parents (Figure 2). The genetic traits exhibited consistency with the principles of autosomal recessive inheritance. In accordance with the ACMG guidelines, the c.1418C > T (p.S473F) variant was classified as a likely pathogenic variant (PM3_Strong + PP3_Moderate + PM2_Supporting). In a patient diagnosed with renal tubular acidosis (24, 25), a potential pathogenic variant was identified at the trans position of the mutation (PM3). REVEL assigned a score of 0.822, suggesting moderate pathogenicity and providing evidence for the presence of deleterious effects on genes or their products (PP3). This variant, absent from the Thousand Genomes Project (1000G) and Shenzhou databases as of August 2024 but present at low frequencies in ExAC (9.06  ×  10^−5^) and gnomAD (4.28 × 10^−4^), fulfills the PM2 criterion (Supporting) for pathogenicity.

**Table 3:** Gene mutation information of the patient.

Gene	Chromosome position	Variation information	Zygotic classification	Genetic pattern	Origin of variability
*ATP6V0A4*	chr7: 13,87,45,183–13,87,45,183	NM_020632.3: c.1418C > T: p.S473F	Subject: Homozygosity	AR	Parent
			Father: Hybrid		
			Mother: Hybrid		

H + -ATPase A4 subunits are expressed not only in type A cells of the kidney but also in the epithelial cells of the inner ear lymphatic sac (9). The vascular stria within the cochlea is not only a high-metabolism tissue but also plays a crucial role in the secretion and absorption of electrolytes in endolymph. This function significantly contributes to the generation of electrical potentials within the cochlea. Cochlear hair cells are rich in lysosomes, where V-H + -ATPase is widely present on the lysosomal membrane surface (20). This enzyme hydrolyzes ATP to transport protons into the lysosome, thereby maintaining its acidic environment (20). Variations in pH may influence the permeability balance of other ions. These genetic abnormalities are frequently linked to advanced deafness and may not necessarily result in hearing loss (9). A study reported a case involving a child with a complex heterozygous mutation in the *ATP6V0A4* gene (c.1418C > T and c.2419C > T) who did not exhibit any hearing impairment (25). A patient with a homozygous mutation in the *ATP6V0A4* gene (deletion of exons 3–5) was monitored until the age of 11, at which point it was observed that the child exhibited hearing impairment, aligning with the characteristics associated with advanced sensorineural deafness (26). This study reports a case involving a child with a homozygous *ATP6V0A4* gene mutation (c.1418C > T) who has been monitored through the early developmental stages up to 10 months of age. To date, no hearing impairment has been observed; however, it is important to note that hearing loss associated with *ATP6V0A4* mutations typically manifests later in life. The limited follow-up period has resulted in the absence of documented deafness, which represents a limitation of this study. Therefore, ongoing dynamic assessment of their long-term auditory status remains essential for the purposes of early detection, timely intervention, and enhancement of quality of life.

In addition to sensorineural hearing loss, the long-term consequences of primary dRTA resulting from variations in the *ATP6V0A4* gene also encompass growth retardation, renal impairment, and kidney failure. With the prompt institution of adequate alkali therapy, the prognosis of dRTA is generally excellent. It was reported that the presence of proteinuria and chronic kidney disease (CKD stage 3a) in a child who was monitored until the age of 11 years; subsequently, the dosage of citric acid mixture was adjusted, leading to a gradual improvement in renal function to CKD stage 2, while proteinuria did not significantly deteriorate (26). Zhao Xiaoying conducted a 5-year follow-up study on a patient receiving chronic oral administration of citron mixture, during which the child's growth and development progressively aligned with normal levels (27). The findings indicated that the judicious application of a citric acid mixture could achieve an optimal growth level while also postponing the onset of renal damage. However, emerging long-term follow-up data indicated a risk of progressive renal impairment in these patients. This was illustrated by one series in which 5 of 10 children with *ATP6V0A4* mutations developed declining renal function, alongside findings from Palazzo et al. that CKD can progress to a detectable stage by adolescence (17, 28).

In this study, children are presently undergoing long-term oral supplementation of potassium citrate. At the onset of the disease, it was observed that the child exhibited secondary hyperaldosteronism and had mildly elevated blood pressure; consequently, oral spironolactone was administered but subsequently discontinued. The observed increase in secondary aldosterone levels may be attributed to the following factors (29). Dysfunction in H+ secretion within the distal renal tubules leads to a reduction in Na + -H+ exchange at the apical membrane of these tubules and an enhancement of Na + -K+ exchange. Furthermore, the insufficient reabsorption of Na+ results in an elevated excretion of Na+ in the urine. Consequently, the renin-angiotensin-aldosterone system is activated, leading to an elevation in secondary aldosterone secretion.

## Conclusion

This study delineates the clinical and genetic features of a pediatric patient diagnosed with primary dRTA attributed to a homozygous mutation in the *ATP6V0A4* gene. It offers a molecular foundation for etiological diagnosis, genetic counseling, and prenatal assessment of dRTA. Timely diagnosis and intervention for primary dRTA can significantly enhance the long-term prognosis of children affected by growth, developmental issues, and renal impairment. We will implement a comprehensive long-term follow-up management plan for this child.

## Data Availability

The datasets presented in this article are not readily available because this article is a case report of a single case and involves privacy issues, the data cannot be shared in public databases. Requests to access the datasets should be directed to the corresponding author.
