# Supplementary material for: A case report and literature review of primary distal renal tubular acidosis resulting from a mutation in ATP6V0A4
Source: Front Pediatr. 2025 Nov 27;13:1685798. doi: 10.3389/fped.2025.1685798 (PMC12696175; doi:10.3389/fped.2025.1685798)
Supplement: Supplementary file 2 [file Table1.docx]

| Mutated Genes | Protein Encoding |
| --- | --- |
| *SLC4A1* | AE1 protein |
| *ATP6V1B1* | the B1 subunit of H+-ATPase |
| *ATP6V0A4* | the A4 subunit of H+-ATPase |
| *ATP6V1C2* | the C2 subunit of V-type H+-ATPase |

**Supplementary Table 1.** Key genes and encoded proteins Involved in dRTA. Abbreviation: AE1, anion exchange protein 1.
